# Supplementary material for: Integrative multi-omics defines melanoma drug response networks and ARID1A-dependent resistance mechanisms
Source: Mol Syst Biol. 2026 Feb 18;22(5):685–711. doi: 10.1038/s44320-025-00183-5 (PMC13144321; doi:10.1038/s44320-025-00183-5)
Supplement: Supplementary file 1 — Appendix [file 44320_2025_183_MOESM1_ESM.pdf]

Appendix

Integrative multi-omics defines melanoma drug response networks and ARID1A-dependent resistance mechanisms

Charlie George Barker[1,2], Sumana Sharma[1,3], Ana Mafalda Santos[3], Konstantinos-Stylianos Nikolakopoulos[4], Athanassios D. Velentzas[4], Cristina Tormo-Garcia[3], Anushka Sharma[3], Franziska I. Völlmy[5], Angeliki Minia[6], Vicky Pliaka[6], Joseph Clarke[3], Maarten Altelaar[5-7], Gavin J. Wright[8], Leonidas G. Alexopoulos[9,10], Dimitrios J. Stravopodis[4], Evangelia Petsalaki[1+]

[1] European Molecular Biology Laboratory, European Bioinformatics Institute (EMBL-EBI), Wellcome Genome Campus, Hinxton, Cambridgeshire, CB10 1SD, United Kingdom
[2] (current address) University College London Cancer Institute, London WC1E 6DD, UK
[3] Radcliffe Department of Clinical Medicine and Medical Research Council, Translational Immune Discovery Unit, Weatherall Institute of Molecular Medicine, University of Oxford, Oxford, United Kingdom
[4] Section of Cell Biology and Biophysics, Department of Biology, School of Science, National and Kapodistrian University of Athens (NKUA), 15701 Athens, Greece
[5] Biomolecular Mass Spectrometry and Proteomics Group, Utrecht Institute for Pharmaceutical Science, Utrecht University, Utrecht, The Netherlands
[6] Netherlands Proteomics Center, Padualaan 8, 3584 CH Utrecht, The Netherlands
[7] Mass Spectrometry and Proteomics Facility, The Netherlands Cancer Institute, 1066 CX Amsterdam, The Netherlands
[8] Department of Biology, Hull York Medical School, York Biomedical Research Institute, University of York, York, UK
[9] Protavio, Demokritos Technology Park, Building 27, Patriarchou Grigoriou & Neapoleos 27, 15341 Aghia Paraskevi, Attiki, Greece
[10] Department of Mechanical Engineering, National Technical University of Athens, Athens, Greece

[+] Correspondence should be addressed to Evangelia Petsalaki
^ Authors contributed equally to this work

Table of Contents

1 Appendix Figure S1. Summary of count information for ’omics data 2
2 Appendix Figure S2. Drug-agnostic network-based changes. 3
3 Appendix Figure S3. Combination therapy-specific network-based changes. 4
4 Appendix Figure S4. Cross-talk in network-based changes between ARID1A-rewired and drug-agnostic response. 5
5 Appendix Figure S5. Summary of melanoma patients extracted from TCGA. 6

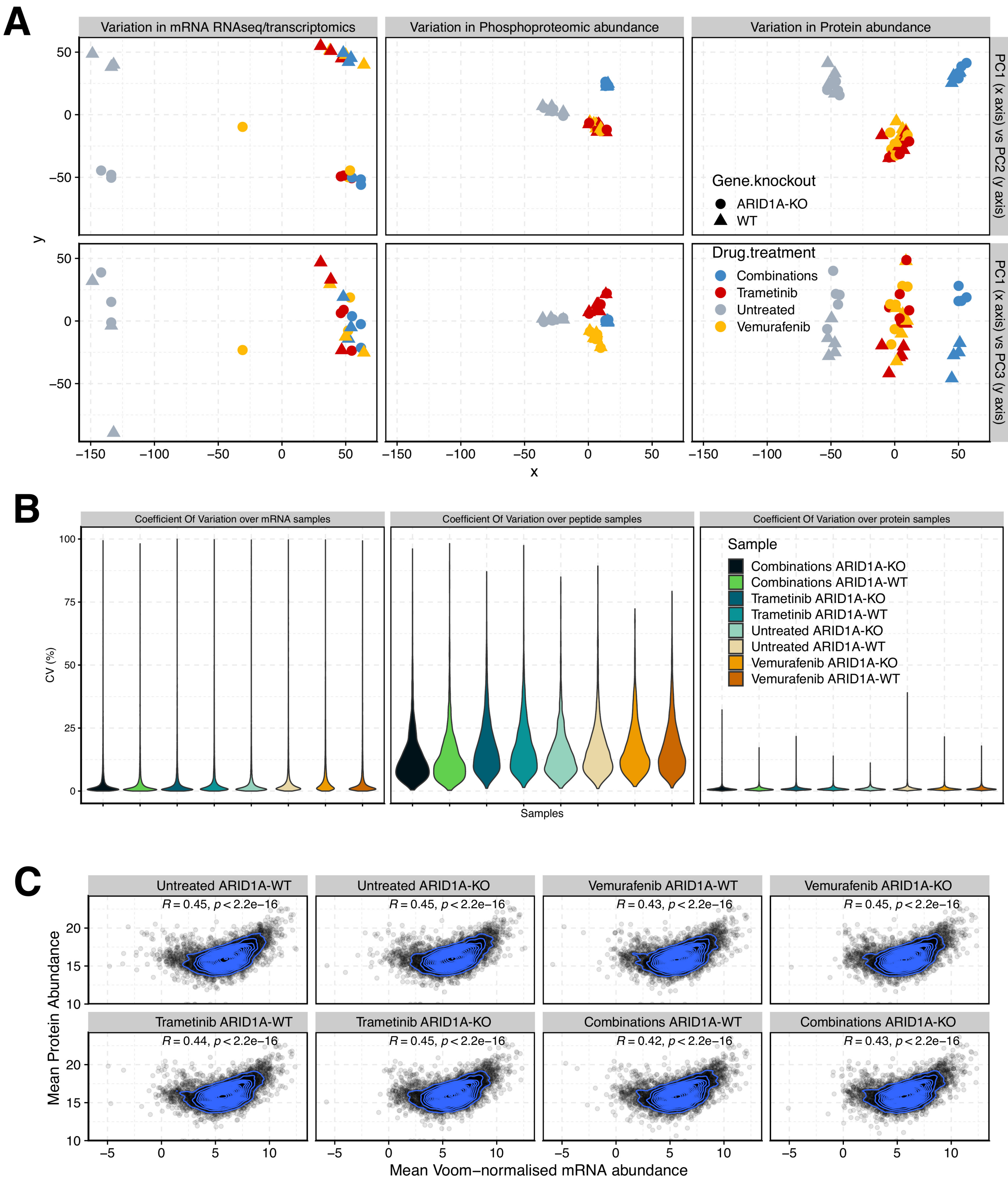

Appendix Figure S1. Summary of count information for ’omics data in different experimental conditions. A. Multidimensional scaling (MDS) for RNAseq (left), peptides (middle) and proteins (right) showing the genetic background (shape) and the drug treatment (colour) experimental conditions. **B.** Coefficient Of Variation (COV) plots for RNAseq (left), peptides (middle) and proteins (right).**C.** Comparison of protein abundance (y axes), versus mRNA abundance (x axes) for different experimental conditions. Statistical correlation was determined using a Pearson’s correlation.

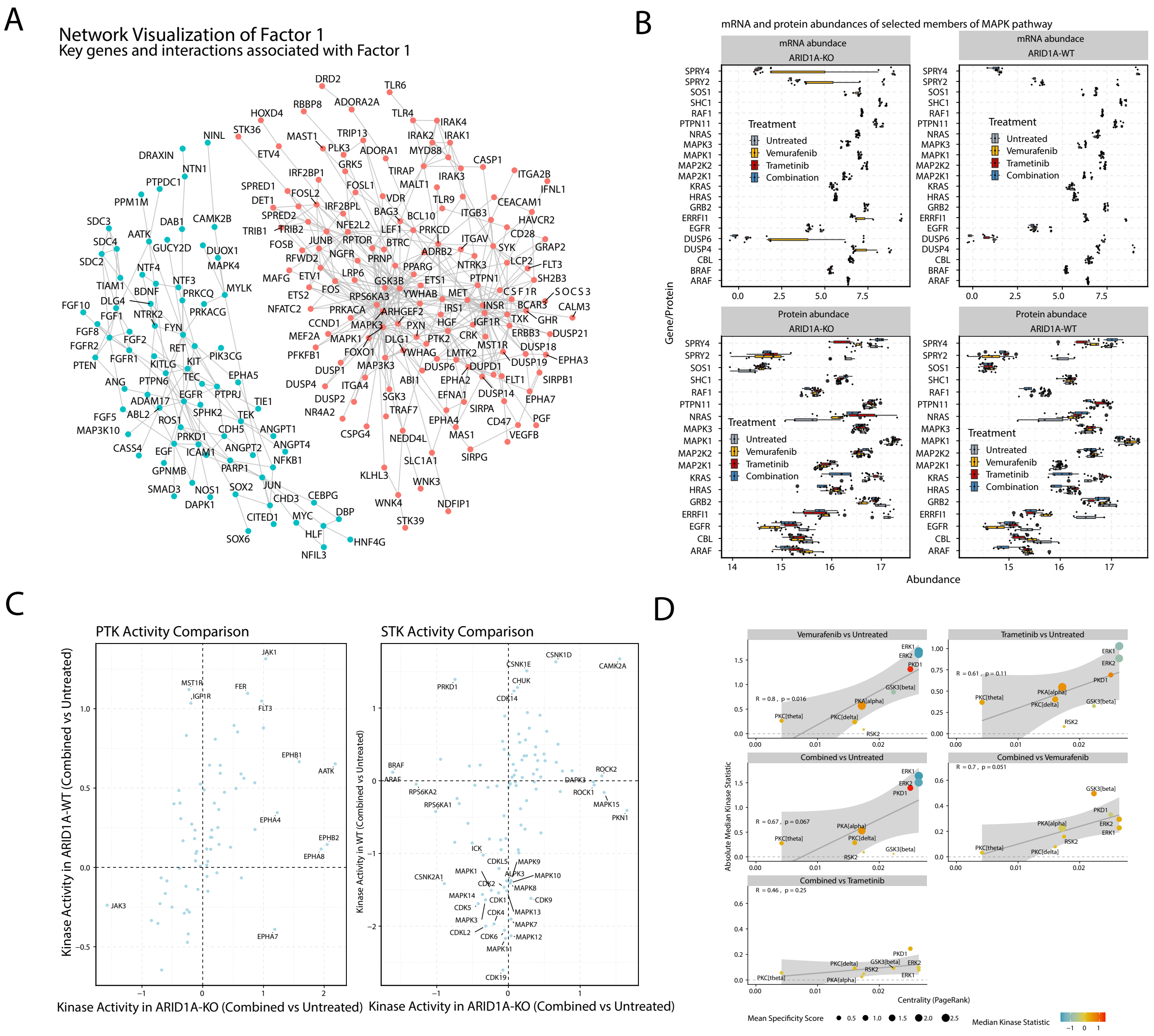

Appendix Figure S2. Drug-agnostic network-based changes. A. PhuEGO-derived Network underlying changes associated with Factor1. Nodes represent proteins and the edges between them represent known interactions between those proteins. Red indicates networks associated with upregulated processes and blue indicates networks associated with downregulated processes. **B.** Changes in abundance (x axes) of MAPK negative feedback regulators (y axes) identified by Gerosa *et al.* (3 samples in each condition). Colour represents the drugs used. Colours represent the drugs used. Boxplots display the median (centre line), the 25th–75th percentiles (bounds of the box), and whiskers extending to the most extreme data points within 1.5×IQR of the quartiles. Outliers beyond this range are shown as individual points. **C.** Kinase activity assay showing the activation of kinases after combined drug treatment in ARID1A-KO (x axes), vs ARID1A-WT (y axes) cell lines.**D.** Absolute values for median kinase activity correlated with centrality of network in EV 5A. Pearson correlation coefficients (r) and corresponding p-values are shown (n=6).

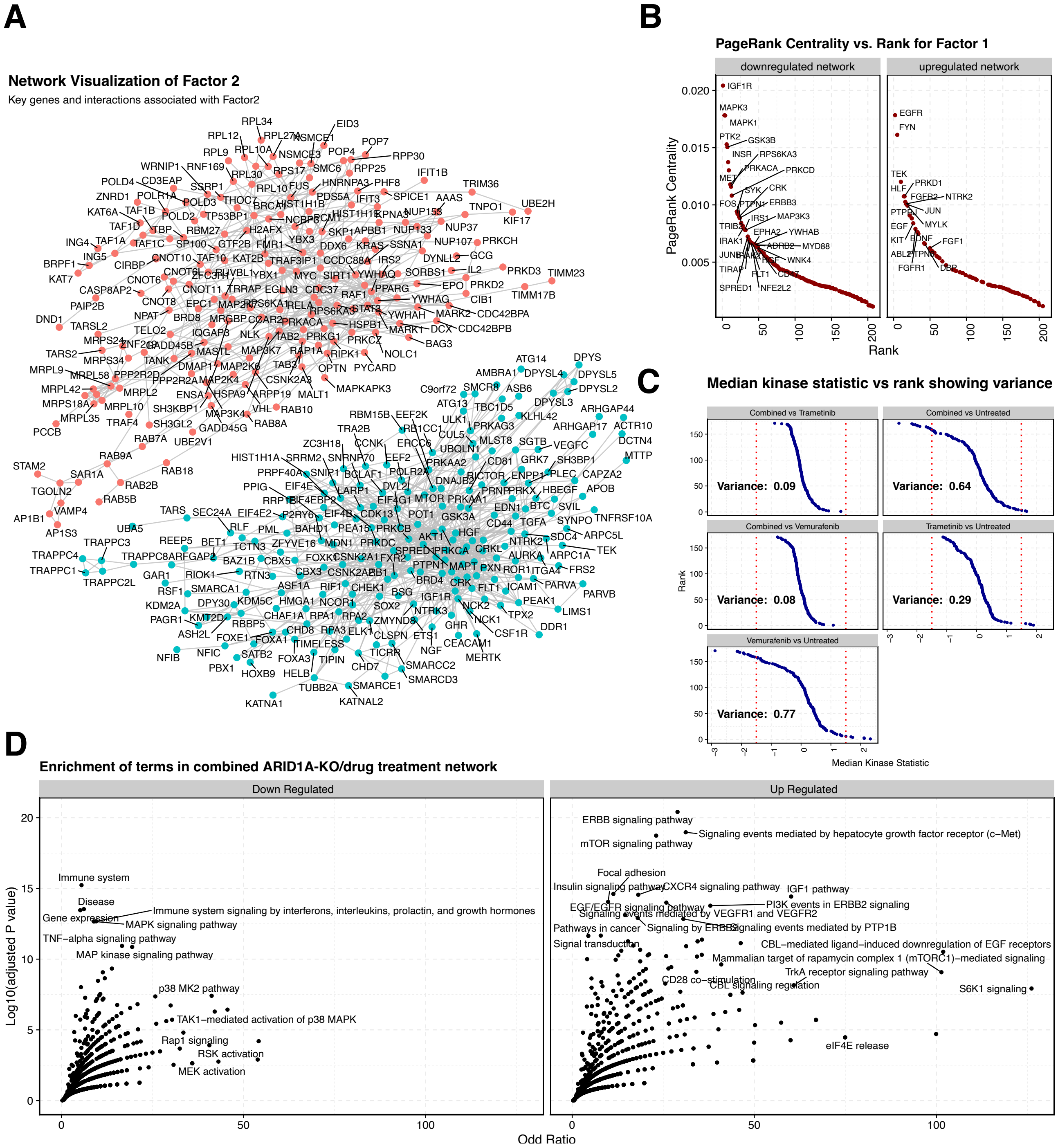

Appendix Figure S3. Combination therapy-specific network-based changes. A. PhuEGO-derived Network underlying changes associated with Factor2. Nodes represent proteins and the edges between them represent known interactions between those proteins. Red indicates networks associated with upregulated processes and blue indicates networks associated with downregulated processes. **B.** Centrality (PageRank, y axis) of nodes in the network represented in Figure EV6A, ranked by order of size (x axes). **C.** Changes in median kinase activity (x axes), showing the difference in variance amongst all experimental conditions. **D.** Functional enrichment analysis of upregulated and downregulated genes in Factor2 derived network, using the pathway database Bioplanet 2019. Axis represent strength of the association (Odds ratio, x axes) and the significance (Log10(FDR adjusted value), y axis).

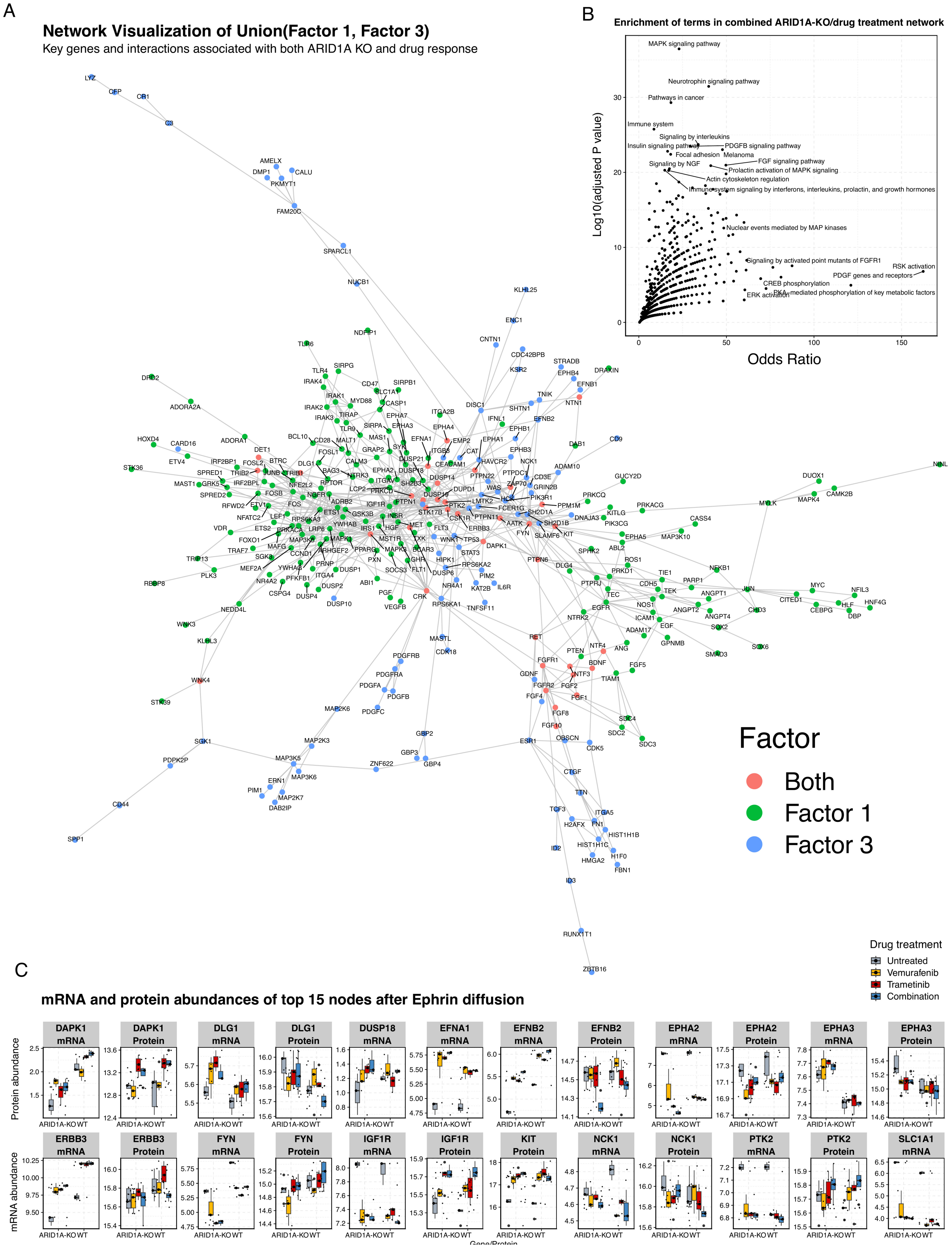

Appendix Figure S4. Cross-talk in network-based changes between ARID1A-rewired and drug-agnostic response. A. Combined network from phuEGO-derived Network underlying changes associated with Factor 3 (blue) and Factor 1 (green). Nodes represent proteins and the edges between them represent known interactions between those proteins. **B.** Functional enrichment analysis of genes in combined Factor 1/3 network, using the pathway database Bioplanet 2019. Axis represent strength of the association (Odds ratio, x axis) and the significance (Log10(FDR adjusted value), y axis). **C.** Changes in abundance (x axes) of downstream effectors of Ephrin identified by maximum flow calculations through the combined Factor1/3 network (n=3). Colour represents the drugs used. Colours represent the drugs used. Boxplots display the median (centre line), the 25th–75th percentiles (bounds of the box), and whiskers extending to the most extreme data points within 1.5×IQR of the quartiles. Outliers beyond this range are shown as individual points.

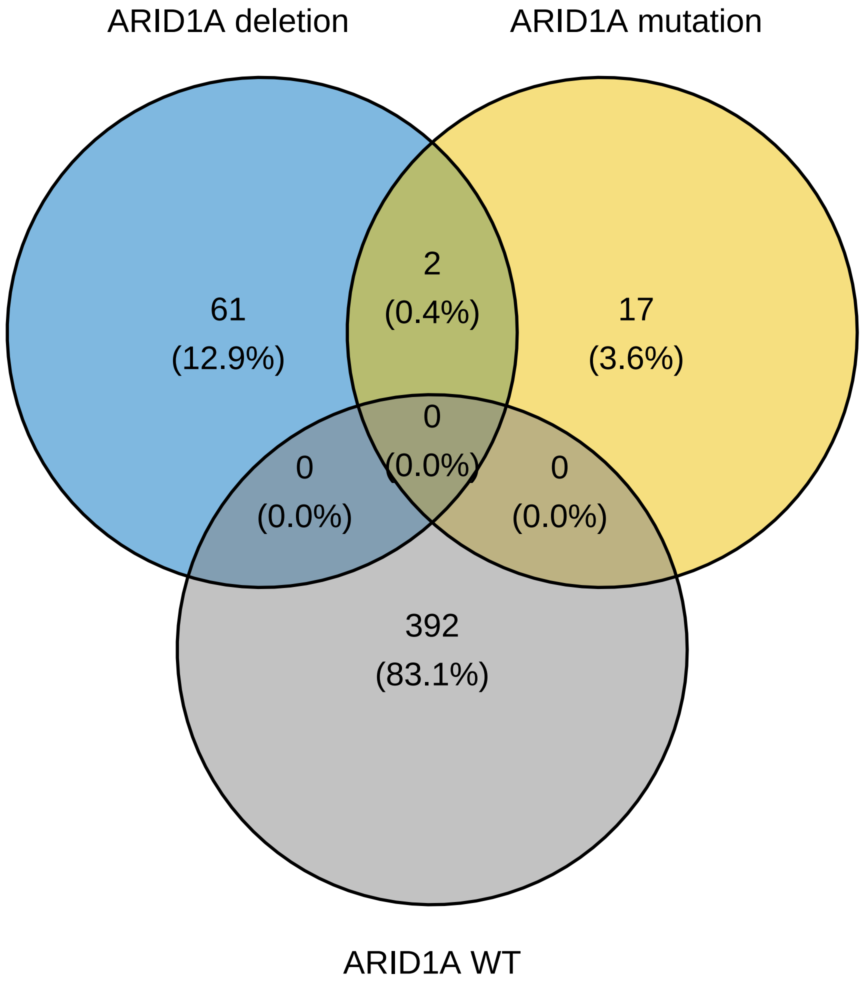

Appendix Figure S5. Summary of melanoma patients extracted from TCGA. Venn diagrams showing patients stratified by whether they have an ARID1A mutation, deletion or neither.
