# Supplementary material for: Integrative multi-omics defines melanoma drug response networks and ARID1A-dependent resistance mechanisms
Source: Mol Syst Biol. 2026 Feb 18;22(5):685–711. doi: 10.1038/s44320-025-00183-5 (PMC13144321; doi:10.1038/s44320-025-00183-5)
Supplement: Supplementary file 16 — Expanded View Figures [file 44320_2025_183_MOESM16_ESM.pdf]

Expanded View Figures

Figure EV1. ARID1A was effectively knocked out and affects signaling dynamics.

(**A**) ARID1A and matched parental melanoma A375 cells were purchased from Synthego. Sanger sequencing traces for the edited region is shown. The single base-pair deletion led to a frame-shift mutation in the protein. (**B**) Output from TIDE analysis showing mismatches in the inference window (left panel) and 98% editing efficiency. (**C**) Killing of parental and ARID1A cell lines in the presence of Vermurafenib, Trametinib, combination of both drugs, and control with DMSO. Starting concentration of 30 nM Vermurafenib and 3 nM Trametinib was used (Left panel). The numbers on x-axis indicate the dilution factor (1:3) from the starting concentration. Images of cells at 90 h post treatment with the drugs (Right panel). (**D**) Luminex-based phosphoprotein measurements of indicated phosphosites on A375 cells treated either with Trametinib (10 nM) for indicated times, or with growth factors (TGFb and EGF) for 4 h. Long term indicates treatment with drug at 1 nM for 2 weeks. Log-fold change is calculated from unstimulated/ untreated condition. Decrease in MEK phosphorylation post trametinib treatment is observed between 1 and 6 h.

© European Molecular Biology Laboratory and the Authors

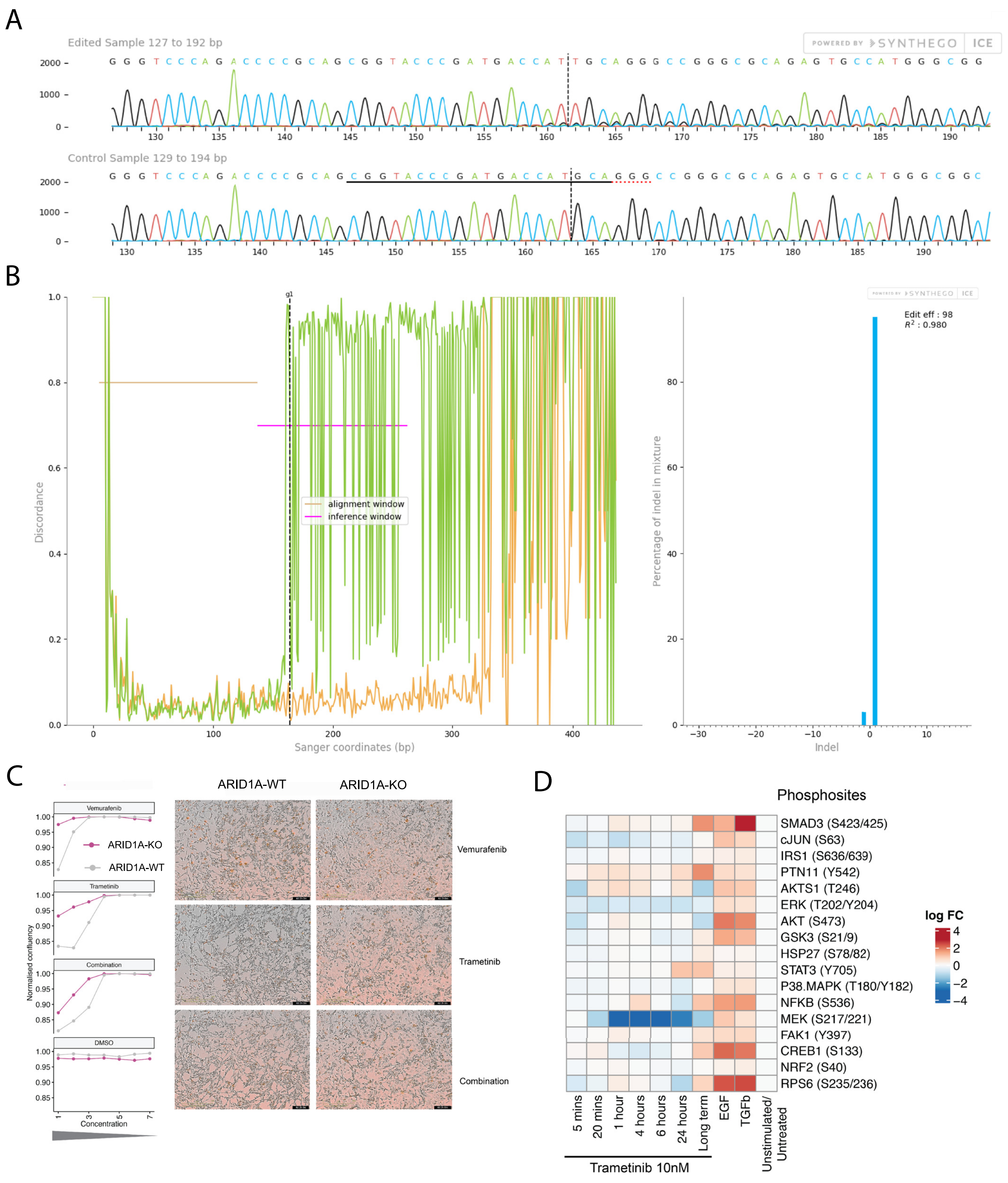
A
Edited Sample 127 to 192 bp
POWERED BY SYNTHEGO ICE
Control Sample 129 to 194 bp
B
g1
alignment window
inference window
Discordance
Sanger coordinates (bp)
Edit eff : 98
R^2 : 0.980
Percentage of indel in mixture
Indel
C
ARID1A-WT
ARID1A-KO
Vemurafenib
Trametinib
Combination
DMSO
Normalised confluency
Concentration
D
Phosphosites
SMAD3 (S423/425)
cJUN (S63)
IRS1 (S636/639)
PTN11 (Y542)
AKTS1 (T246)
ERK (T202/Y204)
AKT (S473)
GSK3 (S21/9)
HSP27 (S78/82)
STAT3 (Y705)
P38.MAPK (T180/Y182)
NFKB (S536)
MEK (S217/221)
FAK1 (Y397)
CREB1 (S133)
NRF2 (S40)
RPS6 (S235/236)
log FC
5 mins
20 mins
1 hour
4 hours
6 hours
24 hours
Long term
EGF
TGFb
Unstimulated/ Untreated
Trametinib 10nM

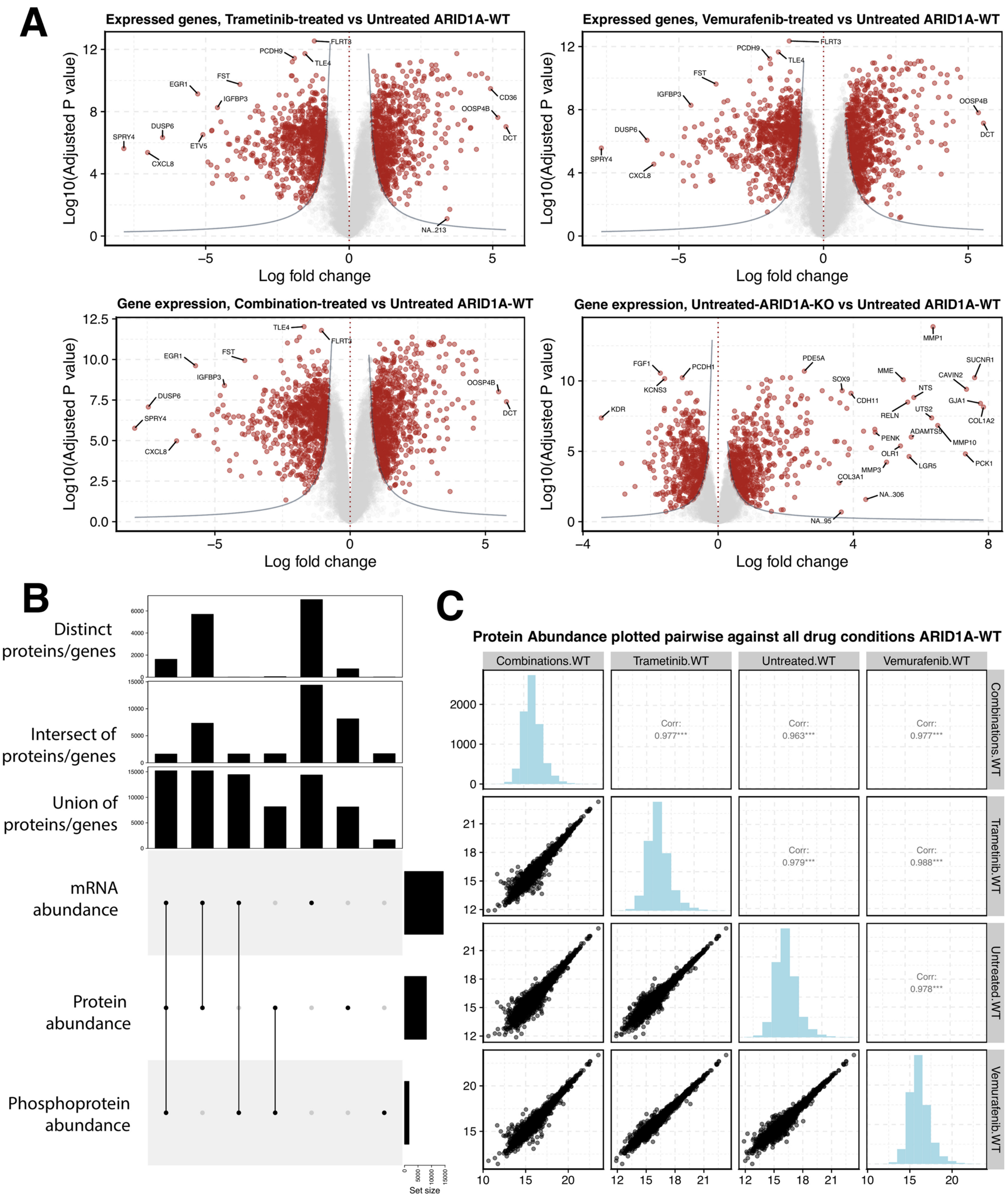

© European Molecular Biology Laboratory and the Authors

Figure EV2. Differential expression analysis and quantified proteins/genes across different 'omics data.

(**A**) Volcano plots for abundant gene transcripts showing significance (FDR-adjusted *P* value, *y* axis) and log-fold change (*x* axis). LFCs for this data as well as phosphopeptides and proteins are available in the EV datasets. (**B**) Upset plot showing the extent of distinct proteins, genes and peptides quantified from each 'omics experiment. Barplots show the number of readings counted in each intersection of the data. (**C**) Plots showing the relation between protein abundance for isolated drug treatments.

© European Molecular Biology Laboratory and the Authors

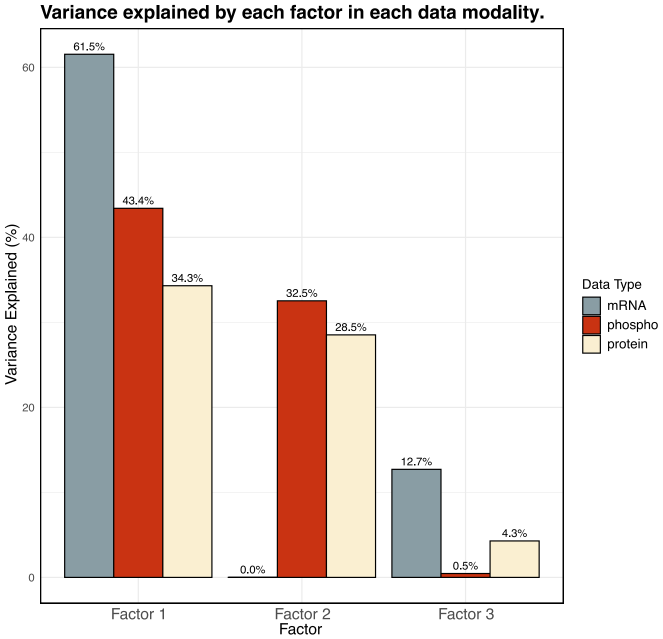

Figure EV3. Variance decomposition derived from MOFA analysis.

Barplot showing the variance decomposition, assessing the proportion of variance explained by each factor in each 'omics modality.

 © European Molecular Biology Laboratory and the Authors

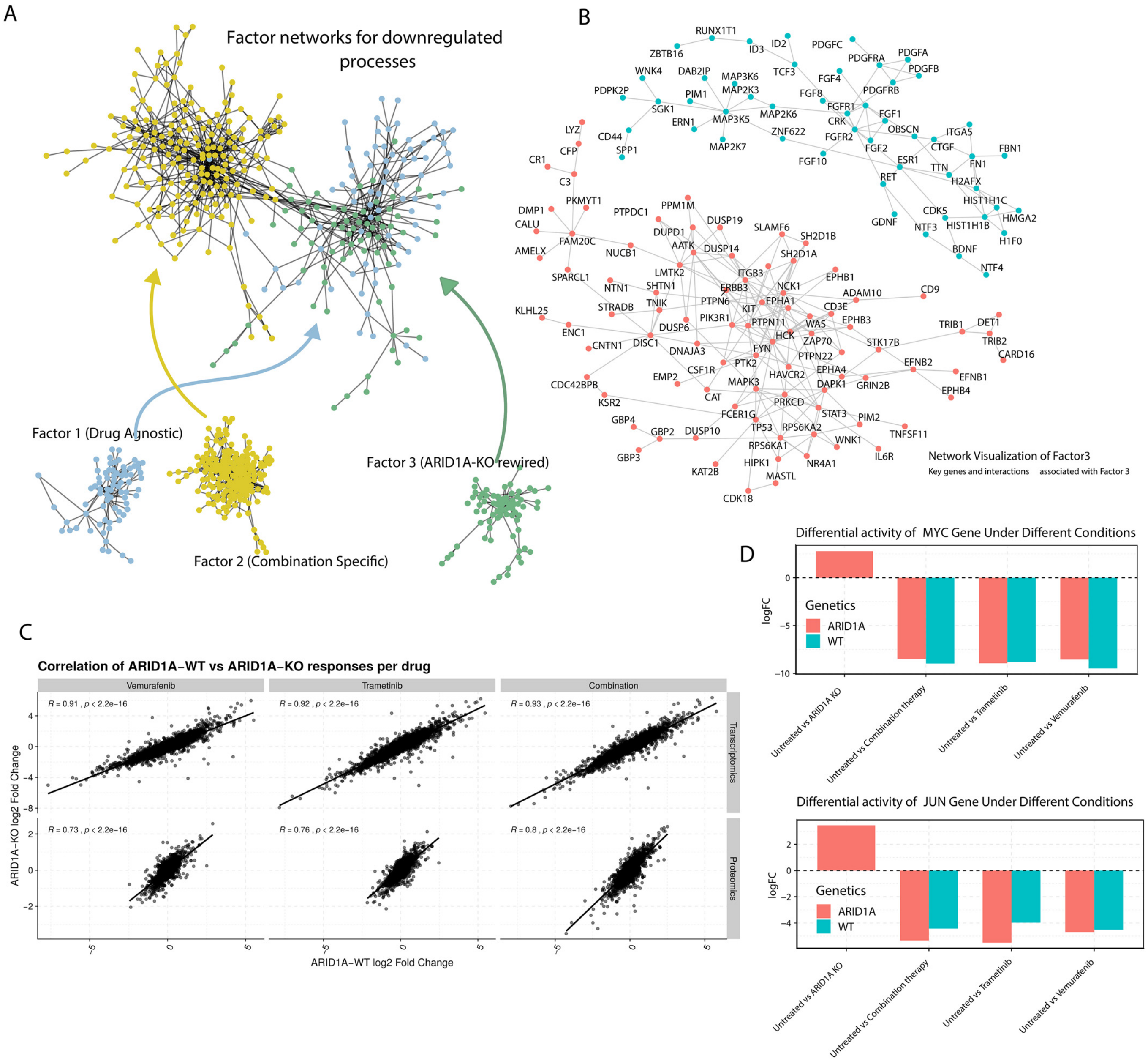

Figure EV4. ARID1A-rewired network-based changes.

(**A**) Graph showing the increased connectivity of nodes from both Factor 1 (blue) and Factor 3 (green) compared to Factor 2 (yellow). (**B**) PhuEGO-derived Network underlying changes associated with Factor3. Nodes represent proteins and the edges between them represent known interactions between those proteins. Red indicates networks associated with upregulated processes and blue indicates networks associated with downregulated processes. (**C**) Scatter plots showing the correlation between ARID1A-KO (*y* axis) and ARID1A-WT (*x* axis) drug response $\log_2$ fold changes in transcriptomic (top row) and proteomic (bottom row) data across different drug conditions (columns). Pearson correlation coefficients (r) and significance values (p-values) were calculated using the Pearson product–moment correlation test, in which significance is assessed using a t-distribution test of the null hypothesis that the true correlation is zero. (**D**) Plot showing the change in activation of TFs MYC (top) and JUN (bottom), based on the relative abundance of their known regulons as calculated using the ColectTRI database. This shows the difference in condition between ARID1A KO A375 cell lines (red) and parental cell lines (blue).

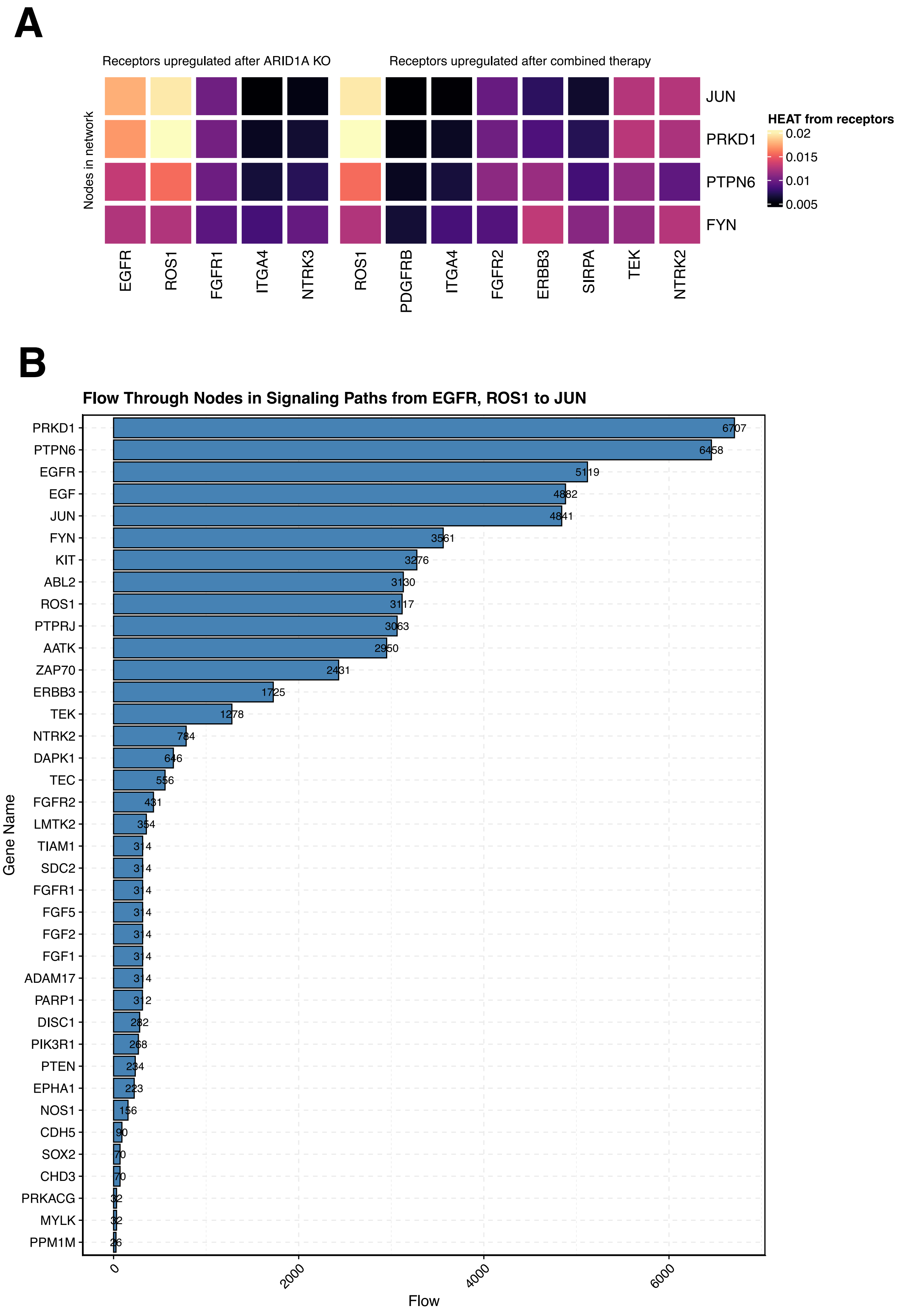

 © European Molecular Biology Laboratory and the Authors

Figure EV5. Network propagation illustrating the effect of dysregulated receptors.

(**A**) Heatmap showing network propagation from individual receptors (columns) and their propagation to selected nodes (rows). (**B**) Barplot showing the flow (*x* axis) through nodes (*y* axis) within the combined Factor1/3 network from EGFR and ROS1 to JUN, ranked in order of flow.

© European Molecular Biology Laboratory and the Authors
